# Thermo-Responsive Antimicrobial Hydrogel for the In-Situ Coating of Mesh Materials for Hernia Repair

**DOI:** 10.3390/polym12061245

**Published:** 2020-05-29

**Authors:** Bárbara Pérez-Köhler, Gemma Pascual, Selma Benito-Martínez, Juan Manuel Bellón, David Eglin, Olivier Guillaume

**Affiliations:** 1 Department of Medicine and Medical Specialties, University of Alcalá, 28805 Madrid, Spain; barbara.perez@uah.es (B.P.-K.); gemma.pascual@uah.es (G.P.); 2 Biomedical Networking Research Centre on Bioengineering, Biomaterials and Nanomedicine (CIBER-BBN), 28029 Madrid, Spain; selma.benito@uah.es (S.B.-M.); juanm.bellon@uah.es (J.M.B.); 3 Ramón y Cajal Health Research Institute (IRYCIS), 28034 Madrid, Spain; 4 Department of Surgery, Medical and Social Sciences, University of Alcalá, 28805 Madrid, Spain; 5 AO Research Institute Davos, Davos 7270, Switzerland; david.eglin@aofoundation.org; 6 3D Printing and Biofabrication Group, Institute of Materials Science and Technology, TU Wien, 1060 Vienna, Austria

**Keywords:** hernia, hyaluronic acid, infection prophylaxis, mesh coating, polypropylene, rifampicin, *Staphylococcus aureus*, thermo-responsive hydrogel

## Abstract

The prophylactic coating of prosthetic mesh materials for hernia repair with antimicrobial compounds is commonly performed before implantation of the mesh in the abdominal wall. We propose a novel alternative, which is a rifampicin-loaded thermo-responsive hydrogel formulation, to be applied on the mesh after its implantation. This formulation becomes a gel in-situ once reached body temperature, allowing an optimal coating of the mesh along with the surrounding tissues. In vitro, the hydrogel cytotoxicity was assessed using rabbit fibroblasts and antimicrobial efficacy was determined against *Staphylococcus aureus*. An in vivo rabbit model of hernia repair was performed; implanted polypropylene meshes (5 × 2 cm) were challenged with *S. aureus* (10^6^ CFU), for two study groups—unloaded (n = 4) and 0.1 mg/cm^2^ rifampicin-loaded hydrogel (n = 8). In vitro, antibacterial activity of the hydrogel lasted for 5 days, without sign of cytotoxicity. Fourteen days after implantation, meshes coated with drug-free hydrogel developed a strong infection and resulted in poor tissue integration. Coating meshes with the rifampicin-loaded hydrogel fully prevented implant infection and permitted an optimal tissue integration. Due to its great performance, this, degradable, thermo-responsive antimicrobial hydrogel could potentially be a strong prophylactic armamentarium to be combined with prosthesis in the surgical field.

## 1. Introduction

Over the last decades, technological advances in the field of reparative medicine and surgery have favored the development of a wide variety of implantable biomedical devices [1]. These biomaterials can be implanted in virtually all the tissue interfaces -either hard or soft tissues- and their utilization allows to reestablish the function of many organs, thus improving the patients’ quality of life [2]. One of the surgical fields in which biomaterials have gained relevant development is the repair of abdominal wall defects, which is a pathological condition that usually manifests hernia development [3]. The global frequency of hernia defects is high, with more than one million of patients being operated yearly in the USA [4]. Among the different surgical approaches available, the implantation of a prosthetic material, so-called mesh, constitutes the gold standard method to repair hernia defects [3].

Despite the obvious benefits of repairing damaged tissues with biomaterials, the implantation of these materials is not exempt of undesired side effects. In this regard, prosthetic mesh infection is considered one of the most devastating post-surgical complications in clinics [5]. In hernia surgery, rates of mesh infection ranges between 1% and 13% depending on several factors such as type of hernia, surgical procedure and device implanted [6]. Patients suffering from mesh-related infections require extended hospital stay and often additional surgeries for debridement and eventually mesh removal, causing a remarkable economic and social impact for the public healthcare systems [7].

To combat this growing problem, there is a need to develop prophylactic strategies aiming at inhibiting bacterial colonization of the implanted mesh. One of the emerging trends is to combine antimicrobial coatings to the mesh to be implanted [8]. This local and controlled administration of drugs reduces the risk of developing bacterial resistance and permits to achieve high concentration of therapeutics in the vicinity of the prosthesis with limited or no systemic effect [9].

The simplest method to coat meshes with antibacterial compounds is performed by soaking the device in antibiotic solutions immediately before its implantation [10], although its local effectiveness is limited given the fast spread of the drug beyond the surgical area. More effective approaches rely on coating the material with polymer compounds exerting antiadhesive, antifouling or antimicrobial properties [11]. The development of bioactive polymers and antibacterial nano-size formulations for biomedical applications are gaining considerable attention given their excellent performance as drug delivery systems [12]. Despite the great outcomes, manufacturing antimicrobial meshes for hernia repair is a costly process that entails the risk of altering the morphological and biomechanical properties of the mesh, which results in a limited clinical translation [9].

A promising alternative to address this shortcoming would be the use of polymer systems exerting the ability to undergo conformational changes in response to a determined environmental stimulus (i.e., temperature, pH, light, etc.). Depending on the type of polymer and the stimulus received, these compounds can undergo precipitation, gelation, exhibit a reversible adsorption on a surface or alternate between hydrophilic and hydrophobic states [13]. Because of their singular properties and the ability to carry and to deliver drugs, stimuli-responsive polymers are considered promising candidates to be used in biomedical applications [14].

We propose to test a new proof of concept, in which the antibacterial coating is applied once the prosthesis has already been implanted into the host tissue. This “in-situ” coating would allow us to cover all the implant with a stimuli-responsive polymer compound loaded with antimicrobials. A thermo-responsive hyaluronic acid-derived hydrogel (HApN) would represent a potential candidate for this purpose. This hydrogel is liquid a room temperature and becomes gel-like above its lower critical solution temperature (LCST). Moreover, it is applicable to numerous mesh materials, can be loaded with a large variety of antibiotics or antiseptics and does not modify the biomechanical features of the meshes [15]. All these points lead us to hypothesize that this singular in-situ coating would bring significant advantages compared to other approaches previously mentioned. To endow the hydrogel with antibacterial activity, rifampicin was loaded in the formulation. This is a liposoluble antibiotic commonly applied to treat device-related infections [16]. Recent in vivo studies evidenced its powerful effect against staphylococcal infections when applied to polypropylene meshes [17,18]. Moreover, rifampicin is currently being used in the manufacturing of prosthetic materials for clinical use [19].

Therefore, the final aim of the present study was to evaluate in vivo the performance of a thermo-responsive rifampicin-loaded hydrogel, which was applied in-situ to coat polypropylene mesh materials following implantation, using a preclinical model of prosthetic hernia repair challenged with *Staphylococcus aureus*.

## 2. Materials and Methods

### 2.1. In Vitro Study

#### 2.1.1. Preparation of HApN Hydrogels and Rheological Characterization

The poly(*N*-isopropylacrylamide) hyaluronan derivative (HApN) was synthetized according to a methodology previously described [15]. Two different hydrogels were prepared by dissolving HApN (10% *w*/*v*) in sterile phosphate buffered saline (PBS; pH 7.4; Sigma-Aldrich, St. Louis, MO, USA) or sterile PBS containing 2 mg/mL rifampicin (Rif; Carl Roth GmbH, Karlsruhe, Germany). These hydrogels were referenced as HApN (drug-free coating) and Rif-HApN (antibacterial coating), respectively.

Rheological measurements were performed to determine the LCST, using an MCR 302 rheometer (Anton Paar GmbH, Graz, Austria) with a Peltier controller with plate-plate geometry (Ø 25 mm). Solutions were subjected to 0.5% oscillatory strain at 1 Hz while heating from 20 °C to 40 °C at a rate of 1 °C/min. The shear moduli (G_0_) of the HApN formulations without and with Rif were recorded as a function of the temperature.

#### 2.1.2. Mesh Coating

The selected mesh was Optilene Mesh Elastic (B. Braun, Melsungen, Germany), a lightweight, monofilament, macroporous polypropylene mesh commonly used in hernia repair. The mesh was cut under sterile conditions into 1 cm^2^ fragments which were subsequently coated with 50 µL of the corresponding HApN or Rif-HApN hydrogel (Rif dose: 0.1 mg/cm^2^) at 4 °C. Once coated, gelation of the hydrogels was triggered by immersion of the meshes into a sterile liquid solution previously warmed at 37 °C.

#### 2.1.3. Release Kinetics

Coated meshes (n = 3) were immersed in 3 mL of warmed sterile PBS and incubated at 37 °C for 5 days. At different time-points, 1 mL aliquots were collected for further evaluation and the same volume of warmed PBS was refilled into each sample. Rif release from coated meshes was assessed using high performance liquid chromatography (HPLC, Agilent Technologies 1260 Infinity with a column Phenomenex “Kinetex 5u C18 100A,” Santa Clara, CA, USA). The mobile phase consisted of a mixture milliQ H_2_O (with 0.2% Trifluoroacetic acid, TFA) with acetonitrile (both from Carl Roth GmbH, Karlsruhe, Germany), ratio 50/50 at a flow rate of 1 mL/min. The Rif was detected using UV-lamp at 345 nm. Results were expressed as the cumulative percentage release of Rif over time.

#### 2.1.4. Cell Culture and Flow Cytometry

Dermal fibroblasts were harvested from skin biopsies collected from the experimental animals, as previously described [20]. Cells were cultured under controlled atmosphere (37 °C, 5% CO_2_, humidity) using Dulbecco’s modified Eagle medium (DMEM) enriched with 10% fetal bovine serum and 1% of antibiotic-antimycotic cocktail for cell culture (Life Technologies, Carlsbad, CA, USA). To assess the cytocompatibility of the hydrogels, fibroblasts were seeded in 6-well plates (2.5 × 10^5^ cells per well) with uncoated meshes or meshes coated with HApN or Rif-HApN (n = 3 each). Following a 24-h incubation, cells were harvested with 0.25% trypsin-EDTA (Life Technologies) and centrifuged at 200 g for 7 min. The resulting pellets were resuspended in 2 mL of flow cytometry buffer (R&D Systems, Minneapolis, MN, USA) and centrifuged again. Cells were resuspended in 400 μL of buffer mixed with 10 μL of propidium iodide (Sigma-Aldrich, St. Louis, MO, USA) and the rate of cell viability was determined using a MACSQuant 10 flow cytometer equipped with a 488 nm laser (Miltenyi Biotec, Bergisch Gladbach, Germany). Data were analyzed using the MACSQuant software provided by the manufacturer (Miltenyi Biotec, Bergisch Gladbach, Germany).

#### 2.1.5. Preparation of Bacteria

*Staphylococcus aureus* ATCC25923 (Sa) purchased from the Spanish Type Culture Collection (Valencia, Spain) was selected to evaluate in vitro and in vivo the antibacterial activity of the hydrogel coatings. An overnight Sa culture at 37 °C in 25 mL of lysogeny broth (LB) (Biomérieux, Marcy l’Etoile, France) was used to prepare a suspension containing a target load of 1.25–1.50 × 10^6^ CFU/mL as described elsewhere [18]. The number of viable Sa was further determined by performing serial dilutions, seeding in LB agar plates and subsequent colony counting, by means of standard microbiological procedures.

#### 2.1.6. Antibacterial Activity of the Hydrogel Coatings

A sequential agar well diffusion test was developed to assess the performance of the hydrogels over time. Uncoated meshes and meshes coated with either HApN and Rif-HApN (n = 3 each) were placed on inoculated LB agar plates (1 mL of a 10^6^ CFU Sa suspension), incubated for 24 h at 37 °C and zones of inhibition (ZOIs) were photographed. Every day, samples were transferred under the same condition on freshly inoculated agar plates until no further ZOI was visualized. To evaluate the ZOI evolution over time, 2 perpendicular diameters per picture were measured using the ImageJ processing software (National Institute of Health, Bethesda, MD, USA).

### 2.2. In Vivo Preclinical Study

#### 2.2.1. Experimental Design and Surgical Technique

Twelve male New Zealand White rabbits weighing approximatively 3000 g were included in the study and the protocol was approved by the University’s Committee on the Ethics of Animal Experiments (referenced as PROEX 045/18). Both surgical procedure and postsurgical monitoring were performed at the University’s Animal Research Center (registration code ES280050001165). The preclinical study was carried out in strict accordance with the National (Spanish Law 6/2013; Spanish Royal Decree 53/2013) and European regulation (European Directive 2010/63/UE; European Convention of the Council of Europe ETS123) on laboratory animal welfare.

Sterile 5 × 2 cm fragments of Optilene mesh were prepared for the preclinical test and randomly distributed to HApN control group (implants coated with drug-free hydrogel, n = 4) or to Rif-HApN group (implants coated with rifampicin-loaded hydrogel, n = 8).

**Figure 1:**
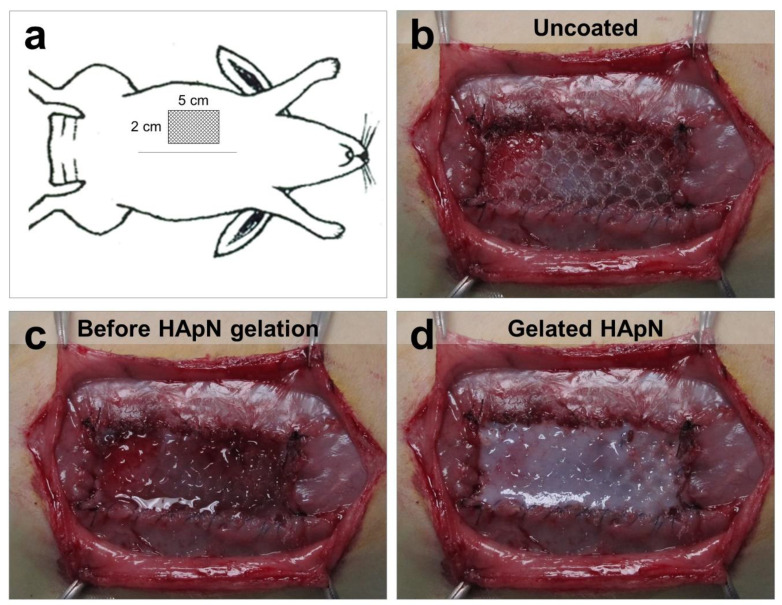
In vivo mesh coating procedure. (**a**) Diagram illustrating the surgical procedure. (**b**) Detail of an implanted mesh. (**c**) Immediately following implantation, the mesh was coated with the corresponding HApN or Rif-HApN hydrogel. (**d**) The hydrogel gelation was triggered using an infra-red lamp for few seconds.

The surgical procedure consisted on the creation of a partial hernia surgical defect followed by mesh repair in preperitoneal position [21]. One hour before surgery and daily during the first 3 postoperative days, each animal received 0.05 mg/kg buprenorphine analgesic (Buprecare; Divasa Farmavic, Barcelona, Spain). A mixture of 20 mg/kg ketamine (Imalgene; Merial, Sant Cugat del Vallès, Spain) and 3 mg/kg xylazine (Xilagesic 2%; Calier, Barcelona, Spain) was intramuscularly administered to induce anesthesia. Then, using a sterile surgical technique, a 5 × 2 cm partial hernia defect was created in the right lateral anterior abdominal wall. The surgical bed was inoculated with 0.25 mL of a 10^6^ CFU Sa suspension prior to placing the biomaterial. All the defects were repaired with the polypropylene mesh, which was secured to the defect edges by running a 4/0 polypropylene suture only interrupted at the implant corners. Once implanted, the mesh was coated with the corresponding hydrogel (50 µL/cm^2^) (Figure 1) and gelation of the coating was immediately triggered by placing an infra-red lamp above the implant for few seconds. Finally, skin tissue was closed by simple interrupted stitches with a 3/0 silk suture.

#### 2.2.2. Postsurgical Monitoring and Euthanasia

Following surgery, animals were regularly weighed and daily monitored to detect any signs of post-surgical complications or infections. Together with the physical inspection, behavior was also tracked to perceive any evidence of animal pain or discomfort. At day 14, the animals were anaesthetized with the ketamine-xylazine mixture previously mentioned and then sacrificed using 20% sodium pentobarbital (Dolethal; Vetoquinol SA, Lure, France).

#### 2.2.3. Macroscopic Observations and Sample Collection

Immediately following euthanasia, implants were visually inspected to evaluate macroscopic outcomes related to infection and tissue repair. The implants plus surrounding tissue were then harvested *en bloc* and subsequently cut into sections (approximate dimensions of 2 × 1 cm) perpendicular to the longest axis of the mesh for further morphologic and microbiologic evaluation.

#### 2.2.4. Antibacterial Effectiveness of the Coated Implants

The bacterial adhesion to the implant surface was quantified after sonicating the biopsies and serial dilution as described elsewhere [18]. For each implant, two tissue samples were sonicated, one harvested from the cranial, lateral side and the other from the central area of the implant. Using a scalpel blade, the mesh was isolated from the host tissue and gently scraped to facilitate bacteria detachment. Host tissue, mesh and scalpel blade were transferred to 20 mL of sterile Neutralizing Pharmacopoeia Diluent (8.5 g NaCl, 2.5 mL Tween-80, 0.35 g lecithin, 997.5 mL distilled water; all reagents from Sigma-Aldrich, St. Louis, MO, USA) and sonicated for 10 min at 40 KHz in a Bransonic 3800-CPXH ultrasonic device (Branson Ultrasonics, Danbury, CT, USA). Then, the suspension was thoroughly vortexed and serially diluted in sterile 0.9% saline for further agar plating, 24-h incubation at 37 °C and colony quantification.

#### 2.2.5. Histology and Immunohistochemistry

Tissue fragments were fixed with F13 solution (60% ethanol, 20% methanol, 7% polyethylene glycol, 13% distilled water; all reagents from Sigma-Aldrich, St. Louis, MO, USA), paraffin-embedded and cut into 5 µm thick sections. Sections were stained with hematoxylin eosin and Masson’s trichrome (Goldner-Gabe variant) to evaluate the mesh integration into host tissue. Then, immunolabeling of Sa was performed to assess the presence and distribution of bacteria throughout the implant. Tissue sections were incubated with the monoclonal antibody against Sa (ab37644; Abcam, Cambridge, UK) in the alkaline phosphatase-labeled avidin-biotin complex (ABC) method and acid hematoxylin was used to counterstain cell nuclei. Both stained and immunostained samples were visualized with an Axiophot light microscope (Carl Zeiss, Oberkochen, Germany).

#### 2.2.6. Scanning Electron Microscopy

Samples were evaluated under scanning electron microscopy (SEM) to qualitatively analyze mesh integration into the host tissue, as well as to determine the presence of any possible signs of infection within the neoformed tissue (i.e., abscess formation, living bacteria).Tissue fragments were fixed with 3% glutaraldehyde, dehydrated in a graded ethanol series (range: 30% to 100%), desiccated with carbon dioxide using a Polaron CPD7501 critical point dryer (Fisons Instruments, Ipswich, UK), coated with gold-palladium and examined in a DSM950 microscope (Carl Zeiss, Oberkochen, Germany).

### 2.3. Statistical Analysis

Mean data collected both in vitro an in vivo were compared between experimental groups using the Mann-Whitney U test implemented in the GraphPad Prism 5 computer package (GraphPad Software Inc., San Diego, CA, USA). The statistical level for significance was set at *p* < 0.05.

## 3. Results

### 3.1. In Vitro Characterization of the Formulated Hydrogels

**Figure 2:**
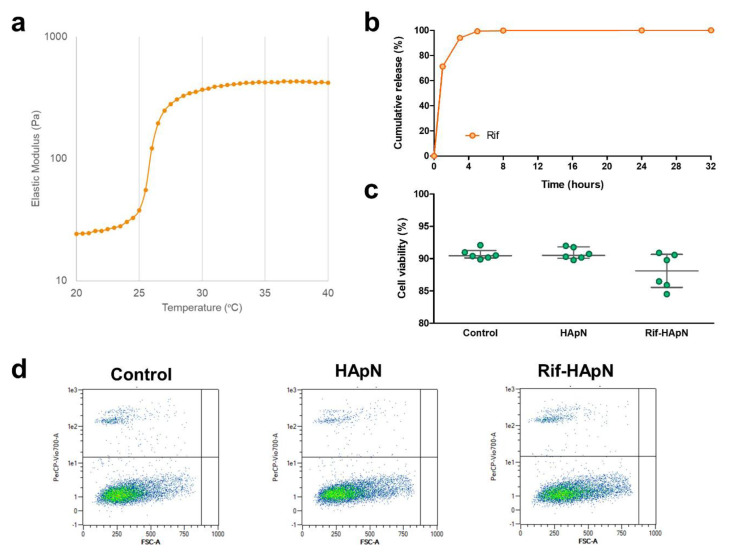
In vitro characterization and cytocompatibility of the hydrogels. (**a**) Rheological assessment of HApN loaded with Rif, showing a lower critical solution temperature (LCST) of 26 °C. (**b**) Cumulative release of rifampicin from 1 cm^2^ polypropylene mesh fragments coated with 50 µL of the Rif-HApN hydrogel (n = 3) and incubated at 37 °C in phosphate buffered saline (PBS). (**c**) Rabbit fibroblasts (n = 6) exhibited high viability after 24 h of culture under exposure to the HApN and Rif-HApN hydrogels. For each group, symbols depict the samples tested and grey lines represent the median and interquartile range. (**d**) Flow cytometry scatter dot plots of the different cell cultures evaluated. For each plot, the x-axis represents the forward scatter cell size (FSC-A) and the y-axis represents the propidium iodide fluorescent signal (PerCP-Vio700-A).

Rheological measurements were performed on HApN formulations without and with Rif, which allowed to determine the LCST of the different solutions. Previously, we reported a LCST of 28 °C for the drug-free HApN hydrogel [15] and the addition of Rif was responsible for a slight decrease of the polymer gelation at 26 °C (Figure 2a).

Results from HPLC revealed that, after 1 h, about 70% of the antibiotic was released from the coated meshes (Figure 2b). This initial burst release was followed by a slower release for the following hours and the samples reached their maximum cumulative release after 32 h.

The in vitro cytocompatibility (Figure 2c,d) demonstrated that fibroblasts were highly viable when cultured in the presence of uncoated meshes. The rates of viability recorded were not significantly different between the uncoated, the HApN and the Rif-HApN groups, thus indicating the absence of any cytotoxicity of these compounds.

**Figure 3:**
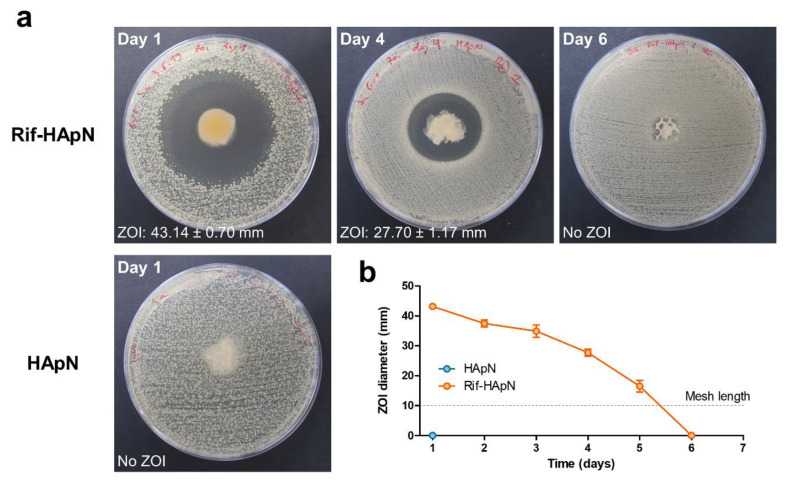
In vitro performance of Rif-HApN against Sa. (**a**) Macroscopic pictures of the zones of inhibition (ZOIs) developed by Rif-HApN in the agar plates at selected time-points (days 1, 4 and 6). As expected, drug-free HApN did not exert any antibacterial activity. (**b**) Measurement of the ZOIs revealed a strong, sustained antibacterial effect of the meshes coated with the Rif-HApN hydrogel (n = 3) that lasted for 5 days.

As revealed by the sequential agar diffusion test (Figure 3), the polymer coating HApN did not produce any antibacterial effect in the absence of drug. However, a strong antibacterial activity was exerted by Rif-HApN. Wide ZOIs were observed for the antibacterial hydrogel for up to three days of incubation. From day 4 onwards, the amplitude of these ZOIs gradually decreased until day 6, where no ZOI was visible anymore.

### 3.2. In-Situ Coating of Implants

Immediately following the surgical procedure, all the meshes were either coated with HApN or Rif-HApN by administering 50 µL of hydrogel per 1 cm^2^ of implant (Rif dose: 0.1 mg/cm^2^, Figure 1). Before gelation, both formulations were slightly viscous and easily infiltrated all the mesh pores plus the suture anchorage site. Once the gelation process was triggered, the hydrogel coating turned firmer and more turbid, creating a smooth and stable layer on top of the implant.

### 3.3. Postoperative Follow-Up

None of the animals deceased as consequence of the surgical procedure or bacterial inoculation. Postsurgical complications like wound dehiscence, mesh displacement, necrosis or edema were not recorded. Postoperatively, visual monitoring revealed a slight skin erythema in two specimens belonging to HApN group that lasted for about 48 h post-surgery. In this group, the development of small bulges under the skin of the animals was confirmed by day 9 and gradually became larger over time. These observations were not recorded in any of the specimens undergoing Rif-HApN coating. Animals from both groups exhibited an expected initial weight loss which was promptly reversed. By day 14, Rif-HApN group displayed a weight gain (7.3 ± 2.1%) compared to HApN group (5.0 ± 2.9%), even though not significant.

### 3.4. Macroscopic Outcomes at Euthanasia

**Figure 4:**
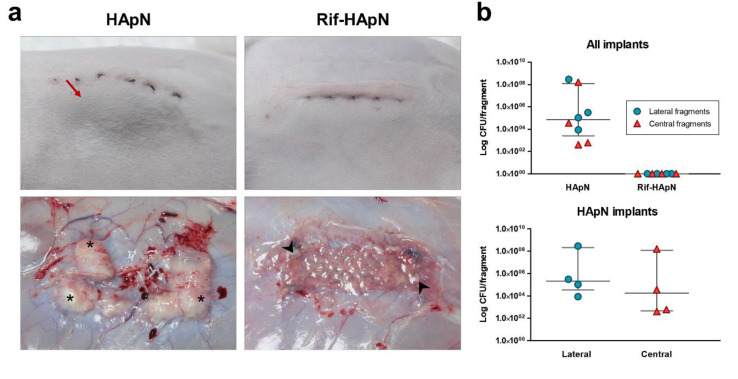
Response of the meshes to infection following 14 days of the bacterial challenge. (**a**) The HApN group evidenced bulges under the skin incision (→) and large amounts of purulent material deposits (*), while Rif-HApN only showed a discrete presence of hydrogel remnant (➤) at the implant margins. (**b**) Sonication of tissue samples yielded no living bacteria in the Rif-HApN implants, while HApN were strongly colonized by Sa. In HApN group, no differences were observed between the bacterial yields from the lateral and central fragments. For each group, symbols depict the samples tested and grey lines represent the median and interquartile range.

The implants coated with HApN were partially encapsulated in a layer of fibrous tissue and the meshes showed noticeable superficial vascularization, although thrombosis was not recorded (Table 1 and Figure 4a). These implants displayed several abscesses covering almost all the mesh surface and the suture anchorage line (4 out of 4 animals). Abscesses correlated with the bulges previously observed under the skin tissue and contained large amount of purulent material. In those areas containing abscesses, mesh was not properly integrated into the host tissue. Contrary to these observations, the surface of the Rif-HApN implants was completely free of abscesses and exhibited milder superficial vascularization. Although some specimens developed fibrous encapsulation, this layer was considerably thinner than the ones from the other study group. The lack of purulent material on the surface allowed the observation of minimal, dispersed remnants of polymer coating at the implant margins, thus confirming the partial biodegradability of this compound.

### 3.5. Bacterial Adhesion to the Mesh

As expected, all the tissue explants collected from HApN implants were colonized by Sa. Overall, bacterial yields were higher in those fragments collected from the lateral margin than those from the central region of the implant, although this difference was not statistically significant (Figure 4b and Table 2. In contrast to these findings, none of the Rif-HApN implants yielded positive counts, revealing absence of Sa adhering to the antibacterial-coated meshes.

### 3.6. Histological Evaluation

**Figure 5:**
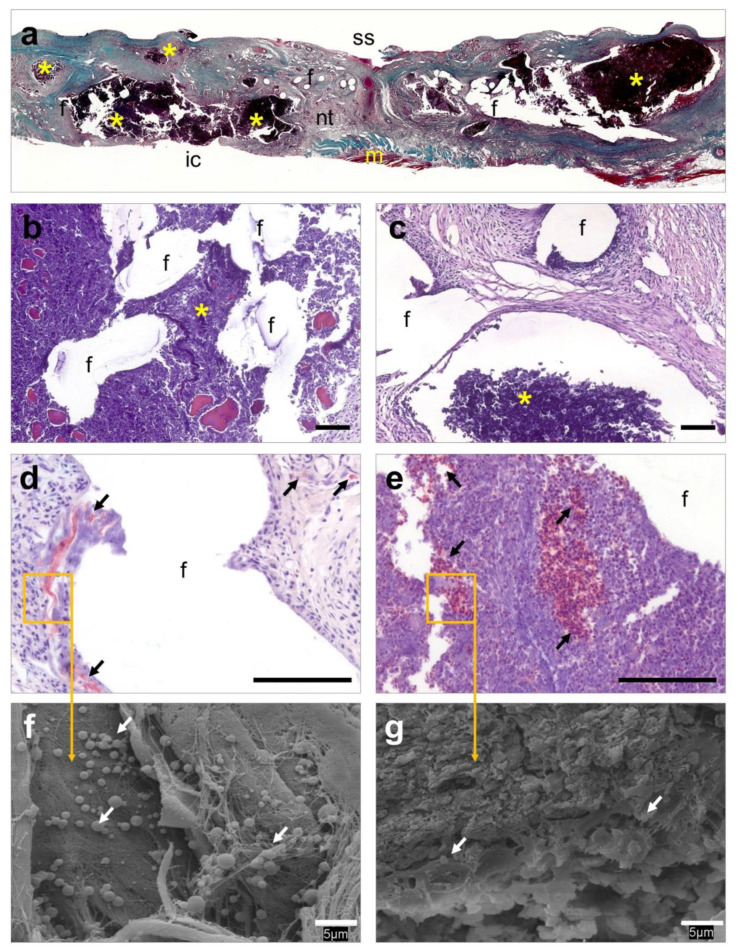
Histological evaluation of the HApN implants. (**a**) Panoramic view of an implant displaying several abscesses that disrupt tissue integration (Masson’s trichrome, ×50). (**b**,**c**) Detail of different areas of the implant showing a dense connective tissue surrounding the mesh filaments, containing different-sized abscesses next to the mesh filaments (hematoxylin eosin, ×100). (**d**,**e**) Presence of bacteria was immunohistochemically confirmed in neoformed tissue and within abscesses (Sa immunostaining, ×320). (**f**,**g**) At higher magnification, bacteria were visualized forming colonies firmly adhering to the implant surface and within the abscesses (scanning electron microscopy, SEM, ×2000). Symbols: (ic) intraperitoneal cavity; (f) mesh filaments; (m) muscle; (nt) neoformed tissue; (ss) subcutaneous side; (*) abscess; (→) bacteria. Scale bars represent 100 µm (b–e) and 5 µm (f,g).

**Figure 6:**
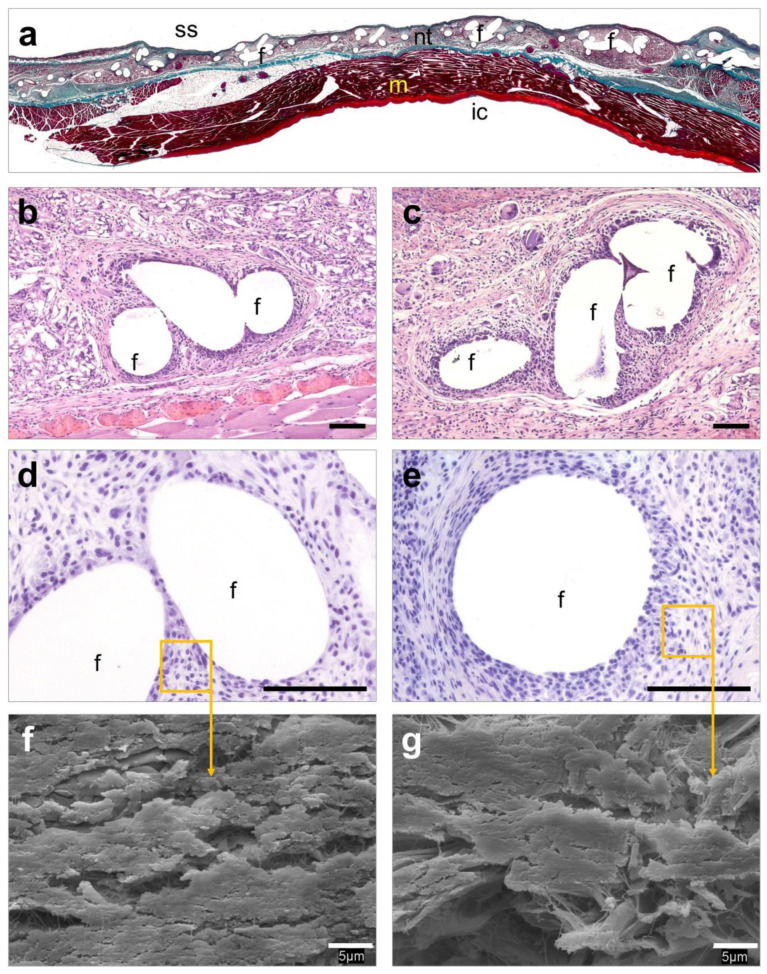
Histological evaluation of the Rif-HApN implants. (**a**) Panoramic view of an implant revealing adequate tissue integration (Masson’s trichrome, ×50). (**b**,**c**) Detail of different areas of the implant showing a loose neoformed connective tissue surrounding the mesh filaments with no evidence of infection (hematoxylin eosin, ×100). (**d**,**e**) No living bacteria were recorded throughout the implant (Sa immunostaining, ×320). (**f**,**g**) Scanning electron microscopy (SEM) visualization confirmed the absence of bacteria in these implants (SEM, ×2000). Symbols: (ic) intraperitoneal cavity; (f) mesh filaments; (m) muscle; (nt) neoformed tissue; (ss) subcutaneous side. Scale bars represent 100 µm (b–e) and 5 µm (f,g).

Implants coated with HApN exhibited numerous abscesses of different sizes which were mainly located in combination with the prosthetic filaments and suture material (Figure 5a). These structures were embedded in a fibrous neoformed connective tissue with a notably impaired mesh integration into the host tissue (Figure 5b). Layers of inflammatory cells and foreign-body giant cells were observed along the tissue (Figure 5c). Immunohistochemical detection of Sa (Figure 5d,e) and further SEM evaluation (Figure 5f,g) revealed the presence of bacteria within the abscesses and in the areas of the connective tissue adjacent to the mesh filaments and sutures. Contrary to these observations, Rif-HApN implants displayed a loose connective tissue which infiltrated the mesh pores in concentric fashion (Figure 6a). Inflammatory cells and foreign-body giant cells were mainly visualized surrounding the mesh filaments (Figure 6b,c). Consistent with the data previously recorded with the sonication assay, no bacteria were found in these implants as confirmed by Sa immunolabeling (Figure 6d,e) and SEM evaluation (Figure 6f,g).

## 4. Discussion

The utilization of in-situ gel-forming antibacterial coating is an appealing approach to control biomaterial-related infections. Compared to commercially available drug-loaded or drug-coated implants, such strategy has numerous benefices, such as higher versatility in terms of drugs and target implants and does not modify important properties such as the biomechanics of the medical devices. This approach can be achieved with the use of the so-called smart polymeric materials; that is, compounds exerting the ability of modifying their physico-chemical properties in response to a specific external stimulus, returning to the original state when the stimulus disappears [13,22]. Among the wide variety of smart polymers available, thermo-responsive compounds have great potential for biomedical applications given their biocompatibility, self-healing and shape memory properties [23]. The ability of thermo-responsive hydrogels to respond to changes in temperature makes them highly effective drug carriers [24,25]. The HApN hydrogel used in this study is easy to store as dry powder and to reconstitute as solution when needed. Upon injection, it can be applied onto implanted prostheses as an in-situ coating or even fill tissue defects of any sizes and shapes. As shown in this work, HApN can also be combined with potent anti-staphylococcal drugs such as rifampicin.

This hydrogel contains poly(*N*-isopropylacrylamide). This is a neutral amphiphilic polymer that dissolves in cold water and undergoes a coil-to-globule transition when heated up above its LCST. Due to the increase of the hydrophobic interactions, this conformational change results in a polymer precipitation and the LCST of poly(*N*-isopropylacrylamide) has been reported to be at around 32 °C. In our case, hyaluronic acid was covalently bound to poly(*N*-isopropylacrylamide) chains and the resulting LCST was found to be close to 28 °C [15]. Hyaluronic acid was selected for its biocompatibility purposes, as it is a well-known natural polysaccharide and a major component of the extracellular matrix in connective tissues. It has been described that LCST of poly(*N*-isopropylacrylamide) in water is not dependent of its molecular weight and concentration but it significantly decreases upon addition of small amounts of hydrophobic compounds or even alcohol [26]. This correlates with our findings as we noticed that adding rifampicin, which is a hydrophobic drug, decreased the LCST from 28 to 26 °C. Nevertheless, this slight change does not limit the range of application of the HApN and the injectable nature of this drug delivery coating provides numerous attractive features.

Other poly(*N-*isopropylacrylamide) hyaluronan derivative polymers were previously applied in vivo with promising outcomes. Ter Boo et al. designed a similar HApN hydrogel loaded with gentamicin and tested its effectiveness using a rabbit model of bone repair with bacterial infection [27,28]. Their findings revealed that the administration of this hydrogel effectively avoided the development of infection without altering the normal process of bone healing. Recently, we evaluated this approach in vitro, demonstrating that the HApN hydrogel can be loaded with either antibiotics or antiseptic agents. Furthermore, its utilization is compatible with several mesh materials of different chemical compositions and architectures [15].

The selected antibiotic, rifampicin, was previously reported as an adequate drug to reduce the risk of infection following hernia repair surgery, where the antibiotic was topically applied onto the implanted mesh [29]. In our study, we proved that the in-situ Rif-HApN coating is not cytotoxic and does not hamper the tissue integration of meshes implanted in the abdominal wall of rabbits. As the formulation remains liquid below the body temperature, it can flow and easily fill complex tissue defects or cover implants with irregular shape, before undergoing gelation. Using a rabbit model of prosthetic hernia repair infected with *S. aureus*, we demonstrated that it is possible to coat an already implanted polypropylene mesh with the hydrogel carrying the antimicrobial agent. Loading 0.1 mg of Rif per cm^2^ of mesh was sufficient to completely eradicate bacteria, as the Rif-HApN coated meshes did not show any sign of infection and no remaining CFU after 14 days of implantation, while minimizing risk of systemic side effect.

Our previous in vitro experiments have shown that other drugs can be loaded in the HApN hydrogel, like gentamicin or even chlorhexidine [15]. Antibiotic and antiseptic-coated meshes are available in clinics for complex abdominal wall hernia repairs with high risk of developing infection (e.g., DualMesh Plus from W. L. Gore & Associates, Inc., Newark, DE, USA, or XenMatrix AB from C. R. Bard, Inc., Murray Hill, NJ, USA) [30,31,32]. Nevertheless, their clinical utilization is extremely restricted due to issues in terms of cost, limited choice of loaded drugs (grafts available with rifampicin and minocycline or chlorhexidine and silver) and limited choice in terms of mesh substrates [9]. Taking those limitations into consideration, we have shown that our HApN might alleviate numerous of those shortcomings and has great potential to be used as versatile and biodegradable antibiotic carrier for hernia meshes.

Results from this study suggest that HApN hydrogels could be relatively easily translated into clinics to prevent postoperative surgical site infection. This prophylaxis is commonly carried out via perioperatively systemic administration of antibiotics, although effectiveness of this strategy following hernia surgery is a matter of debate [33]. In some specific cases, that is, clean laparoscopic surgeries, the risk of provoking undesired side-effects or bacterial resistances makes systemic administration of drugs a non-recommended approach [34]. In those cases, the in-situ coating of meshes with a hydrogel carrying the target antibiotic that can be locally applied on the implant represents an interesting alternative to the systemic antibiotic prophylaxis.

Together with the prevention of postoperative infection, these antibacterial polymers could have a promising application to treat as well already established device-related infections. Those severe cases often require the removal of the prostheses, as conservative approaches are not always successful due to the absence of viable local or systemic antibiotherapy [35,36].

In this regard, a future perspective of this work would be to assess the utilization of HApN in a scenario of established mesh infection.

## 5. Conclusions

We have developed a thermo-responsive and degradable smart hydrogel which can be used to coat hernia meshes directly after their implantation in the abdominal wall. Combining this hydrogel with rifampicin allowed to endow meshes with strong anti-staphylococcal activity, both in vitro and in a preclinical condition. Further work will assess the possibility to use this Rif-HApN to eradicate established mesh-related infections, which management remains frequently a huge challenge for many surgical teams.

## Figures and Tables

**Table 1:** Macroscopic outcomes of the different implants. A compiled scoring is provided, summarizing the most relevant macroscopic outcomes from each study group at euthanasia.

Macroscopic Outcomes Relative to Infection		
Outcome	HApN (n = 4)	Rif-HApN (n = 8)
Skin necrosis	0	0
Fistula	0	0
Edema	0	0
Abscess with purulent material	1 (>50% implant surface) 3 (<50% implant surface)	0
**Macroscopic Outcomes Relative to Tissue Repair**		
	HApN (n = 4)	Rif-HApN (n = 8)
Superficial vascularization	2 (Moderate) 2 (Severe)	6 (Normal) 2 (Moderate)
Thrombosis	0	0
Encapsulation	3 (Moderate to thick) 1 (Very thick)	2 (Non-encapsulated) 6 (Thin to moderate)
Mesh integration	4 (Partial integration)	8 (Complete integration)

**Table 2:** Bacterial adhesion to the surface of the different implants. Quantification of bacterial adhesion to the implant surface via sonication. Results are described as the average bacterial load (CFU) yielded from the lateral and central fragments collected from the implants of the different study groups.

	HApN		Rif-HApN	
Value	Lateral (CFU)	Central (CFU)	Lateral (CFU)	Central (CFU)
Minimum	8.60 × 10^3^	4.00 × 10^2^	0	0
25% Percentile	3.30 ×10^4^	4.50 × 10^2^	0	0
Median	2.05 × 10^5^	1.78 × 10^4^	0	0
75% Percentile	2.16 × 10^8^	1.20 × 10^8^	0	0
Maximum	2.88 × 10^8^	1.60 × 10^8^	0	0
Mean	7.21 × 10^7^	4.00 × 10^7^	0	0
Std. Deviation	1.44 × 10^8^	7.99 × 10^7^	0	0
